# *Drosophila melanogaster* as a model to study innate immune memory

**DOI:** 10.3389/fmicb.2022.991678

**Published:** 2022-10-20

**Authors:** Marta Arch, Maria Vidal, Romina Koiffman, Solomon Tibebu Melkie, Pere-Joan Cardona

**Affiliations:** ^1^Tuberculosis Research Unit, Germans Trias i Pujol Research Institute (IGTP), Badalona, Spain; ^2^Department of Genetics and Microbiology, Universitat Autònoma de Barcelona, Bellaterra, Spain; ^3^Comparative Medicine and Bioimage Centre of Catalonia (CMCiB), Germans Trias I Pujol Research Institute (IGTP), Badalona, Spain; ^4^Microbiology Department, Laboratori Clínic Metropolitana Nord, Germans Trias i Pujol University Hospital, Badalona, Spain; ^5^UCBL, UnivLyon, Université Claude Bernard Lyon 1 (UCBL1), Villeurbanne, France; ^6^Centro de Investigación Biomédica en Red en Enfermedades Respiratorias (CIBERES), Instituto de Salud Carlos III (ISCIII), Madrid, Spain

**Keywords:** *Drosophila melanogaster*, innate immune memory, trained immunity, tolerance, resistance, infection

## Abstract

Over the last decades, research regarding innate immune responses has gained increasing importance. A growing body of evidence supports the notion that the innate arm of the immune system could show memory traits. Such traits are thought to be conserved throughout evolution and provide a survival advantage. Several models are available to study these mechanisms. Among them, we find the fruit fly, *Drosophila melanogaster*. This non-mammalian model has been widely used for innate immune research since it naturally lacks an adaptive response. Here, we aim to review the latest advances in the study of the memory mechanisms of the innate immune response using this animal model.

## Introduction

The immune system can be generally divided into an innate and an adaptive arm (Medzhitov and Janeway, 2000). For long, the dogma in immunology has been that the innate immune response is rapid and non-specific, while the adaptive immune response is slower, but antigen-specific and led to long-term immunological memory. However, it is unlikely for a crucial trait like immune memory to be restricted to the adaptive arm of the immune response when more than 95% of species do not rely on this immune system and many studies have provided pieces of evidence that some vaccines and infections protect against secondary exposure in a specific or unspecific way (Hirano et al., 2011; Conrath et al., 2015; Reimer-Michalski and Conrath, 2016). Up to date, several models have been proposed for studying innate immune memory, such as plants, vertebrates, and invertebrates (Netea et al., 2011, 2020).

The fruit fly *Drosophila melanogaster* has been recognized as an outstanding model to study host-pathogen interactions and immunity (Lemaitre and Hoffmann, 2007; Buchon et al., 2014; Galenza and Foley, 2019; Aromolaran et al., 2021). Over the years, several infection models have been evaluated in the fruit fly, so a deep understanding of the molecular mechanisms taking place in the host after infection has been gained [Extensively reviewed lately in Younes et al. (2020) and Michael Harnish et al. (2021)].

In this review, we have first compiled the latest insights regarding the host’s immune response to consecutive challenges and the subsequent acquisition of innate immune memory. Secondly, we outlined the relevant characteristics that make of *D. melanogaster* a good model for understanding innate immune memory and the methods available to assess it both orally and systematically.

## Innate immune memory

### Definition and mechanisms of innate immune memory

An increasing number of evidences that infections or exposure to microbial-derived compounds can induce not only specific protection against reinfection but also non-specific protection against a subsequent challenge with another pathogen have been described in mice models (Di Luzio and Williams, 1978; Krahenbuhl et al., 1981; Muñoz et al., 2010; Marakalala et al., 2013; Ribes et al., 2014). It has also been described that vaccination with bacille Calmette-Guerin (BCG) conferred protection against a range of infectious diseases in both mice (Van’t Wout et al., 1992; Kaufmann et al., 2018; Covián et al., 2019; Witschkowski et al., 2020) and humans (Garly et al., 2003; Aaby et al., 2011; Biering-Sørensen et al., 2017; Rieckmann et al., 2017; Arts et al., 2018; Walk et al., 2019) as well as induced antitumoral effects through the activation of innate immune cells like monocytes and macrophages (Villumsen et al., 2009; Stewart and Levine, 2011; Buffen et al., 2014; Redelman-Sidi et al., 2014). The non-specificity of this protection, the shortened and reversible protective effect (Nankabirwa et al., 2015; Netea et al., 2016; Dominguez-Andres and Netea, 2019) and the evidence that plants and invertebrates, which only rely on innate immunity, also showed greater protection against reinfections (Dimopoulos et al., 2002; Moret and Siva-Jothy, 2003; Rodrigues et al., 2010; Norouzitallab et al., 2016; Reimer-Michalski and Conrath, 2016; Melillo et al., 2018; Lanz-Mendoza and Contreras-Garduño, 2022) revealed that immunological adaptation may also occur on the innate immunity.

The nomenclature regarding the innate immune memory acquisition process is still controversial. We describe below the basic concepts used in this field and how they have evolved as a result of deepening their study (also listed in Box 1). The concept of “trained immunity” was first proposed by Netea et al. (2011), being referred as a long-lasting functional reprogramming of innate immune cells after exposure to a microorganism or an inflammatory stimulus that lasts over time and leads to an altered response toward a second challenge. Nevertheless, this reprogramming has also been seen in epithelial stem cells, showing faster mobilization and higher induction of interferon-stimulated genes during secondary challenges (Naik et al., 2017, 2018). The first exposure of the host to the challenge is defined by the authors in the field as priming (Cooper and Eleftherianos, 2017; Sheehan et al., 2020). As perfectly detailed in the review by Bindu et al. (2022), the innate immune reprogramming happening after priming is given *via* epigenetic and metabolic modifications of trained cells which can lead either to an increase or a decrease in immunity (Boraschi and Italiani, 2018). The mechanisms of this reprogramming are not completely understood yet, though some evidence supports the existence of multiple regulators (Ghisletti et al., 2010; Smale et al., 2014; Fanucchi et al., 2019; Natoli and Ostuni, 2019). In addition, new studies have also suggested changes in DNA methylation patterns and changes in cellular metabolism as a mediator of trained immunity (Donohoe and Bultman, 2012; Cheng et al., 2014; Arts et al., 2016; Verma et al., 2017; Das et al., 2019; Penkov et al., 2019).

**Box 1. Innate immune memory related terms.**

**Table:** 

Trained innate immunity/Innate immune memory	Long-lasting functional reprogramming of innate immune cells after exposure to a microorganism or an inflammatory stimulus that leads to an altered response toward subsequent challenges (Netea et al., 2011, 2016).
Priming	Phenomenon of memory induction which consists of the exposure of dead microbes or sublethal doses of live pathogens to the host in order to activate the innate immune response (Cooper and Eleftherianos, 2017; Sheehan et al., 2020).
Innate immune reprogramming	Transcriptional and epigenetic modifications, accompanied and regulated by induced metabolic changes, happening in innate immune cells when exposed to stimulus (Boraschi and Italiani, 2018; Bindu et al., 2022).
Non-specific acquired resistance/Trained potentiation	Priming-induced enhancement of the innate immune response to subsequent challenges (Boraschi and Italiani, 2018).
Trained tolerance	Priming-induced downregulation of the innate immune response to subsequent challenges (Boraschi and Italiani, 2018).

RNA interference (RNAi) was recently put in light as an important immune priming mechanism which confers an adaptive antiviral response (Tassetto et al., 2017). This mechanism is based on a post-transcriptional gene regulation mechanism by which small interfering RNAs (siRNAS) induce the sequence-specific degradation of homologous messenger RNA (mRNA) sustaining the antiviral effect along time after first exposure (Meng and Lu, 2017). In addition to the cell-autonomous immunity conferred to the infected cell, this antiviral RNAi signals have shown its ability to spread systematically conferring innate immune memory (Tassetto et al., 2017). This antiviral mechanism and its priming capacity have been reported in vertebrates, invertebrates and plants, although the spreading mechanisms differ between taxa (Meng and Lu, 2017; Tassetto et al., 2017; Cooper and Van Raamsdonk, 2018; Gourbal et al., 2018; Javdat and Tamara, 2020).

**FIGURE 1:**
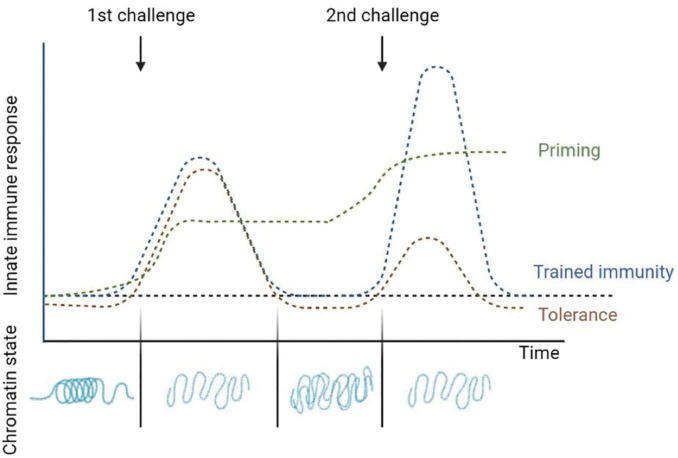
Changes after the induction of innate immune memory. Priming is characterized by an activation of gene expression in innate immune cells that is sustained over time and does not return to basal levels before the second challenge. Often the response to the second stimulus is synergistic with the first one. In trained immunity the gene expressions levels return to basal levels when the first stimulus is removed, but epigenetic changes persist favoring a faster and higher immune response in subsequent infections. Finally, tolerance is the opposite to trained immunity, where after activation by a first stimulus and returning to basal levels the immune response is reduced in subsequent challenges. The flat dotted line represents the basal activation levels of the innate immune response in absence of infection. Adapted from Divangahi et al. (2020) and Netea et al. (2020).

Despite this, the term trained immunity is also used by various authors to refer to the immune phenotype the cells obtain after a primary challenge which allows them to react quicker and stronger to secondary one, thus producing an increase in the immune response to a stimulus (Netea et al., 2020); instead, other authors suggest more accurate terms which describe this phenomenon such as “non-specific acquired resistance,” “potentiation,” or “trained potentiation” (Boraschi and Italiani, 2018). On the other hand, although this reprogramming of innate immunity to an inflammatory profile provides a great advantage in host defense, it may also be detrimental in the context of chronic inflammatory diseases. As compensatory mechanism it has also been shown the opposite reaction, being the priming-induced downregulation, which results in a reduced response after a secondary stimulus (Cooper and Eleftherianos, 2017; Boraschi and Italiani, 2018; Uribe-Querol and Rosales, 2020). Authors will be referring as “tolerance” or “trained tolerance” to this also considered innate memory phenomenon (Boraschi and Italiani, 2018). Tolerance is also induced and maintained by epigenetic changes. Analysis of this mechanism revealed that lipopolysaccharides (LPS) administration could induce alterations in the chromatin that silenced pro-inflammatory genes but not antimicrobial effector genes (Seeley and Ghosh, 2017; Hajishengallis et al., 2019; Lajqi et al., 2021; Figure 1).

Whether adaptation of innate immune cells will enhance or reduce the immune response after a second challenge will depend on the dose and the duration of the first stimulus as well as on the kind of stimuli which not necessarily has to be the same source in both challenges (Divangahi et al., 2020). Based on the functional status of these cells before the second challenge, Divangahi et al. (2020) published a review with an updated definition of the different adaptive programs a cell can incorporate. In addition to trained immunity and tolerance, in which the immune genes expression return to basal levels after the first stimulus is removed and the response after the second challenge is increased or decreased, respectively, these authors also define differentiation and priming as two adaptive programs affecting a cell innate immune response. The change of an immature cell into its mature counterpart is defined as cell differentiation and often comes together with morphological changes; although differentiation can occur during homeostatic conditions, differentiated cells can be trained as well after infection or vaccination (Lavin et al., 2014). On the other hand, when the first stimulus changes the state of the innate immune cells and the active gene expression does not return to basal levels before the second challenge it is named “priming,” and often the response to the second stimulus is synergistic with the first one (Divangahi et al., 2020; Figure 1).

### Resistance and tolerance as consequences of innate immune memory

Upon infection, there are two major strategies to fight disease: resistance and tolerance. Relating these two types of host’s responses to the adaptive programs exposed by Divangahi et al. (2020) trained immunity and priming would relate to an increased host resistance to reinfection, and tolerance would refer to a tolerogenic host response to reinfection. In this regard, resistance strategies will aim at killing the pathogen or inhibit its proliferation (Boraschi and Italiani, 2018). However, mounting an immune response is energetically costly, leading to a reduction in nutrient storage, growth, and reproduction in the organism. This suggests a strong link between immunity and host fitness (Stearns, 1989; Schwenke et al., 2016). The health status of a host does not always depend on the number of pathogens it can bear. Instead, it depends on the ability to reduce the effects of the damage and stress (Chow and Kagan, 2018). Tolerance strategies will reduce the negative effect on fitness caused by the infection, but will not have an impact on pathogen fitness (Howick and Lazzaro, 2017). Since the mechanisms of disease tolerance do not reduce pathogen load, they should promote the transmission of the infectious agent in the population. Some pathogens like *Salmonella enterica typhimurium* induce tolerance to increase their spread. However, throughout evolution, reducing infections, tolerance and resistance mechanisms became highly interconnected (Howick and Lazzaro, 2017). For example, by increasing host fitness, tolerance responses will aim at reducing tissue damage and thus, allowing the resistance mechanisms to work more potently (Martins et al., 2019). Besides, by increasing host fitness, tolerance mechanisms give time to induce resistance strategies to eliminate the pathogen (Chow and Kagan, 2018; Sheehan et al., 2020). Disease tolerance can also be important in cases where the immune response is the one generating the damage, for example, in the case of sepsis (Chow and Kagan, 2018). In some situations, the activation of one pathway can contribute to tolerance and resistance mechanisms. Autophagy, for example, can reduce pathogen burden, thus contributing to disease resistance. However, in other contexts, it can contribute to disease tolerance by reducing endothelial barrier destruction (Chow and Kagan, 2018). Evidence suggests that different organs have different tolerance capacities since they can be more susceptible to damage or have a higher tissue replication rate, allowing a higher reparation rate. Moreover, the consequences of tissue damage can vary according to the tissue. For instance, damages in the endothelium can compromise vascular integrity and lead to ischemia and tissue necrosis, whereas skin damage may not be life-threatening (Medzhitov et al., 2012).

## *Drosophila melanogaster* as a model for studying innate immune memory

### *Drosophila melanogaster* innate immune response

The use of *D. melanogaster* as a model has provided a huge insight into the mechanisms of action of the innate immunity, as insects rely solely on this type of response thus avoiding the variability that adaptive mechanisms imply. In addition, *D. melanogaster* presents a high degree of conserved features with vertebrates including immune cascades, signal transduction pathways, and transcriptional regulators (Younes et al., 2020), as well as a significant amount of well-conserved homologs of disease-causing genes in humans (Bier, 2005). These facts make the fruit fly a good model for studying innate immune responses and gene functionality in basic research.

**FIGURE 2:**
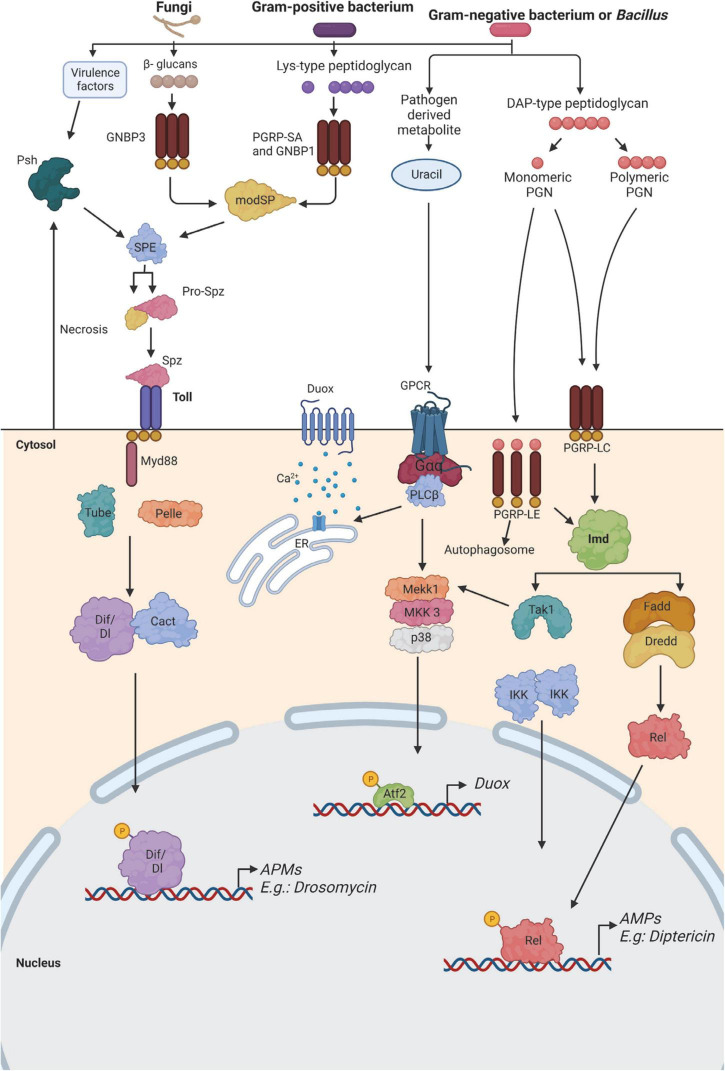
Immune recognition of microorganisms in *Drosophila*. The two major pathways that sense bacteria and fungi in fruit flies are the Toll pathway (left) and the immune deficiency (Imd) pathway (right). Both pathways function in the fat body for the production of antimicrobial peptides (AMP) by activating the expression of NF-κB-like factors, which are highly conserved among species. In addition, the Imd pathway also functions in the epithelial surfaces. The Imd pathway is activated when DAP-type peptidoglycan from gram-negative bacteria, and some gram-positive, binds to Peptidoglycan Recognition Proteins (PGRPs), and this activation leads to the generation of AMP and synthesis of Duox enzyme for the production of reactive oxygen species (ROS). Gram-positive bacteria contain lys-type peptidoglycan, which is recognized by PGRP-SA and Gram Negative Bacteria Protein (GNBP) 1, and GNBP3 binds to β-glucans of yeasts and fungi, leading to the activation of the Toll pathway. This pathway can also be triggered by danger signals like proteases or abnormal cell death that activates the protease Persephone (Psh). In all cases, the activation of the Toll pathway triggers a proteolytic cascade that activates the protease Spätzle- processing enzyme (SPE). This protein will cleave Spätzle (Spz). As a result of the activation of the Toll pathway, the transcription factors Dorsal-related immunity factor (Dif) or Dorsal (Dl) will translocate to the nucleus, thus inducing the expression of AMP genes like *drosomycin*. Similarly, the activation of the Imd pathway induces the nuclear translocation of the transcription factor Relish (Rel) and induction of the expression of AMP genes, such as *diptericin*. The generation of ROS is induced by the activation of Duox in the presence of uracil. This is caused by the activation of a G protein-coupled receptor (GPCR), which promotes the release of calcium from the endoplasmic reticulum. This signaling pathway, together with the activation of the Imd pathway, contributes to the expression of the Duox enzyme during infection. Atf2, activating transcription factor 2; Dredd, death-related ced-3/Nedd2-like caspase; Fadd, FAS-associated death domain ortholog; Gαq, G protein αq-subunit; IKK, inhibitor of NF-κB kinase; MKK3, MAPK kinase 3; modSP, modular serine protease; Tak1, TGFβ-activated kinase 1. Adapted from Buchon et al. (2014) and Younes et al. (2020).

As in vertebrates, *D. melanogaster* immune system is also divided into humoral and cellular responses. The main mode of action of the humoral response in *D. melanogaster* is the production of antimicrobial peptides (AMPs). After a systemic infection, AMPs are released into the hemolymph where they persist for several days and can protect the flies against a second exposure to the pathogen (Boman et al., 1972). AMPs can act synergically or be highly specific depending on the pathogen (Imler and Bulet, 2005; Hanson et al., 2019, 2022; Hanson and Lemaitre, 2020). This response is mediated by the fat body, which is the equivalent of the liver in mammals and represents the main immune-responsive organ in the fly (Lemaitre and Hoffmann, 2007). Barrier epithelial cells are also able to secrete AMPs and reactive oxygen species (ROS) in response to a localized infection (Tzou et al., 2000). Besides this, lower levels of AMPs are also expressed from the hemocytes, muscles, Malpighian tubules, and neuronal tissues (Charroux and Royet, 2009; Badinloo et al., 2018). Three main signaling pathways have been described to play a role in the regulation of immune genes induced after infection. The Toll and the immune deficiency (Imd) pathways regulate the majority of immune genes, including the production of AMPs. The Toll pathway is activated in response to the detection of lysine (Lys)-type peptidoglycan of Gram-positive bacteria, and β-glucans of yeasts and fungi, as well as through the sensing of danger signals, like microbial proteases, or abnormal cell death. On the other hand, the Imd pathway is activated through the detection of diaminopimelic acid (DAP)-type peptidoglycan from Gram-negative and certain Gram-positive bacteria (Buchon et al., 2014; Figure 2). The study done by Kutzer and Armitage (2016) revealed that the Imd and Toll pathways have different kinetics since the AMPs triggered by those pathways were observed at different times after infection. An infection of flies with a gram-negative bacterium such as *E. coli* that triggered the Imd pathway showed the highest AMP concentration after 6 h. In contrast, when challenged with a gram-positive bacterium like *Lactococcus lactis*, thus triggering the Toll pathway, the peak of AMPs was observed 24 h post-infection. These differences in immune pathways contribute to the differences in the clearance of different pathogens (Kutzer and Armitage, 2016). In addition, a different pattern of activation is also observed between males and females (Duneau D. F. et al., 2017; Belmonte et al., 2020). Finally, *Drosophila* also has a complete yet more simple JAK/STAT signaling pathway, which is involved in diverse biological processes, including early and late development, innate immunity, germ-cell adhesion, and inhibition of apoptosis (De Gregorio et al., 2001; Boutros et al., 2002). This pathway also contributes to the immune response by inducing the transcription of thioester-containing protein genes and Turandot stress genes, which, both play a role in *Drosophila* defense against pathogens (Lagueux et al., 2000; Agaisse et al., 2003; Dostálová et al., 2017). In addition, a communication axis between the humoral pathway Imd and JAK/STAT which controls fly antiviral immune response has been recently described (Shen et al., 2022).

Cell-mediated innate immunity of *Drosophila* comprises of immune blood cells, which can be found freely circulating in the hemolymph or associated with diverse tissues. These cells, which are collectively called hemocytes, can be differentiated according to their morphology and immunological functions in plasmatocytes, lamellocytes and crystal cells (Lanot et al., 2001; Hartenstein, 2006; Hultmark and Andó, 2022). Recently, a new class of cells have been identified in *Drosophila*, the primocytes, whose functions are not fully elucidated, but they are thought to regulate the larval hematopoietic organ which, in turn, controls the hematopoiesis of other hemocytes (Hultmark and Andó, 2022). In addition, the presence and proportion of these cells may vary depending on the developmental stage the animal (Honti et al., 2014). Phagocytosis represents a fundamental process of the innate immune response and in the maintenance of tissue homeostasis. In *D. melanogaster* this process is mainly performed by plasmatocytes, which represent up to 90% of the total circulating hemocytes (Wang et al., 2013) and appear to perform the functions of vertebrates’ macrophages and neutrophils (Stramer et al., 2005). Melanization is another predominant immune response in insects based on the production and release of melanin around intruding microorganisms (Christensen et al., 2005). This response is involved in wound healing and hemolymph coagulation, and is mediated by the crystal cells (Ashida and Brey, 1995; Söderhäll and Cerenius, 1998; Tang, 2009; Leitão et al., 2020; Younes et al., 2020). Lastly, lamellocytes are differentiated from plasmatocytes when the cell-mediated innate immune response is induced upon infection with parasitoid wasp, wounding or artificially by genetic induction, and are in charge of the encapsulation of foreign bodies that are too large to be phagocytosed, as well as melanization (Kounatidis and Ligoxygakis, 2012; Honti et al., 2014; Dudzic et al., 2015; Vlisidou and Wood, 2015; Leitão et al., 2020).

In *Drosophila*, the mechanisms by which a septic infection causes a systemic response controlled by the fat body have been well-characterized, even though these types of infections are rare. However, although the so-called natural infections take place constantly, the epithelial immune response remains less characterized. It is known that this local response is mainly regulated by the Imd-pathway (Onfelt Tingvall et al., 2001) and is shaped against different microbes *via* the JAK/STAT and the JNK signaling pathways (Wagner et al., 2009). In 2013, a microarray analysis performed by Gendrin et al. (2013) showed that the induction of Imd-dependant genes varies substantially among tissues with only very few “universal genes” being expressed in the fat body, the gut, and the trachea (Gendrin et al., 2013). This set of genes includes mainly AMPs and pathway components.

Specifically in the gut, local immunity includes physical and chemical barriers as well as a cellular response (Buchon et al., 2013). ROS are induced by two enzymes: dual oxidase (Duox) is stimulated through pathogen-derived uracil and peptidoglycan (Lee et al., 2013) and NADPH oxidase (Nox) is induced by microbiota-derivate lactate (Iatsenko et al., 2018). In the gut, Duox-derived ROS are mainly involved in immune response and repair tissue damage, while Nox-derived ROS regulate epithelial renewal (Iatsenko et al., 2018). However, excessive production of ROS is ultimately toxic to the host and induces epithelial cell death and early ageing, thus Duox expression is tightly regulated through the p38 mitogen-activated protein kinase–activating transcriptional factor 2 (p38 MAPK–Atf2) pathway (Ha et al., 2009b). The uracil secreted by some pathogenic bacteria activates the phospholipase Cβ (PLCβ), which in turn activates the p38MAPK-Atf2 pathway. The PLCβ pathway also promotes the release of Ca^2+^ from the endoplasmic reticulum to bind and activate Duox (Ha et al., 2009a). In response to commensals, the levels of both uracil and peptidoglycan are lower and the concentration of cytosolic Ca^2+^ is reduced, thus Duox activity is kept minimal (Ha et al., 2009b).

**FIGURE 3:**
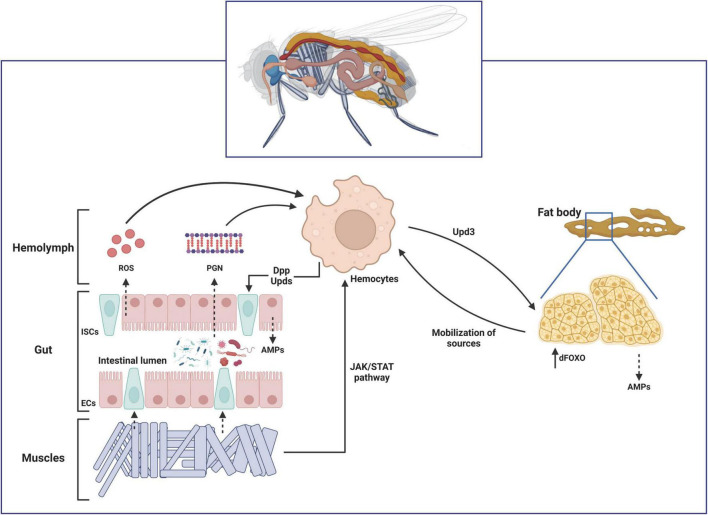
Schematic representation of the interorgan communication during infection in *Drosophila melanogaster*. In response to ingested pathogens, these microorganisms might either break the gut epithelium themselves or release peptidoglycan (PGN) from their cell walls into the hemolymph. The enterocytes (ECs) are also able to release reactive oxygen species (ROS) in response to tissue damage. These circulating signals are sensed by the hemocytes located close to the gut epithelium. Hemocyte-expressed Decapentaplegic (Dpp) and Unpaireds (Upds) during early stages of the infection activate the intestinal stem cells (ISCs) proliferation to cope with tissue damage. Hemocytes-derived Upds also induce accumulation of the transcription factor dFOXO in the fat body *via* JAK/STAT pathway. Activation of dFOXO induces expression of AMPs and a systemic metabolic dysregulation that mobilizes energy resourced toward phagocytic cells. Finally, infection-induced Upd3 in enterocytes activate the JAK/STAT signaling pathway in visceral muscles (VM), which in turns promote ISCs proliferation, as well as activation of the close-located hemocytes.

Several studies have proved that an interorgan communication occurs in *D. melanogaster* by proving that local intestinal infections are able to induce a systemic response in the fat body (Basset et al., 2000; Foley and O’Farrell, 2003; Wu et al., 2012). The simplest explanation would be that when an ingested pathogen is able to break the intestinal epithelial barrier and enter into the hemolymph, circulating immune cells phagocyte them and activate a systemic immune response in the fat body, as observed for the bacteria-derived peptidoglycan (PGN) (Charroux et al., 2018). However, some studies revealed Ecc15 is not detectable in the hemolymph of larvae after ingestion, thus proving that it is unlikely that the bacteria directly interact with the hemocytes in the hemolymph (Basset et al., 2000; Foley and O’Farrell, 2003). Although the specific mechanisms involved have not been extensively explored yet, it has been shown that gut-derived ROS might interact with circulating hemocytes and regulate diptericin production in the fat body cells (Amcheslavsky and Ip, 2012). Other studies also showed a correlation between gut-expressed PGRP-LE with the levels of AMPs expression in the fat body (Paredes et al., 2011; Bosco-Drayon et al., 2012). Finally, a cross-talk between muscles and immunity *via* JAK/STAT activation has also been described (Yang and Hultmark, 2016). This interorgan communication mechanisms have been recently extensively reviewed (Liu and Jin, 2017; Figure 3).

### Methods to assess innate immune memory in *Drosophila melanogaster*

Several methods have been developed that aided in the understanding of the processes related to innate immune memory in the *D. melanogaster* model. According to the research question and the experimental design, different kinds of infection models could be used. For inducing a systemic infection there are several methods available; the most used ones are needle pricking and injection pumping or microinjection (Troha and Buchon, 2019). Needle pricking involves dipping a needle into a solution containing the pathogen, which is later used to prick anesthetized flies. Inoculation can be done in the abdomen or thorax, yet minimal damage should be generated. Since a wound is produced, infection is first local and later disseminates systemically, allowing to assess both types of infections. This type of inoculation is quick; hence this method can be used for the infection of large amounts of flies. However, a limitation of this method is that the infection dose can be variable.

In addition, to be able to observe dissemination and systemic infection, very small number of bacteria should be inoculated by needle pricking to minimize the effect of the local infection (Apidianakis and Rahme, 2009). Therefore, some authors agree that needle pricking would be most appropriate injection method for highly virulent bacteria such as *Pseudomonas aeruginosa* and *Staphylococcus aureus* (Apidianakis and Rahme, 2009; Lee et al., 2018). In case a more precise dose is needed, it is suggested to use the microinjection method. Here, a needle is charged with a specific volume of pathogen solution, and using a pumped injector, the solution is introduced in the abdomen or thorax, entering directly into the haemolymph, thus generating a systemic infection. Although more reproducible, this method is slower and requires specific equipment. Assessing the circumstances, an oral infection could be desirable. Unlike the injecting methods, feeding results in local infection primarily in the gut epithelium (Lee et al., 2018). However, it should be noted that in oral infections the infective dose is also a limitation, as it is variable and difficult to monitor (Troha and Buchon, 2019).

In order to assess innate immune memory acquisition, flies have to be primed by exposing them to either a sublethal dose of a pathogen, a heat-killed pathogen or a pathogen-derived material previously to the second challenge (lethal infection) (Sheehan et al., 2020). Priming can also be performed both orally and systematically, making sure in the last one that both challenges are done one on each side of the fly body (Kutzer et al., 2019). The protective effect is then assessed by comparing survival and pathogen loads between primed and non-primed flies in response to the second challenge.

Other methods to assess the immunological mechanisms behind the innate immune memory acquisition in *D. melanogaster*, include analysis of gene expression of AMPs by RT-qPCR, RNAi, or RNA-seq; protein levels quantitation, phagocytic or melanization activities and release of ROS upon infection (reviewed in Troha and Buchon, 2019). Measuring expression levels of AMPs together with survival could bring surprising results, as it has been observed by several authors that on certain occasions, there were no changes in survival after oral administration of non-pathogenic bacteria, yet levels of AMPs were increased (Patrnogic et al., 2018). In addition, on a genetic level, the process of innate immune memory is thought to be associated with epigenetic changes in the chromatin. To evaluate such changes, methyl marks in histone H3 can be measured, as well as the concentration of enzymes associated with chromatin remodeling, like histone lysine methyltransferases (Bonnet et al., 2019).

Last but not least, several studies have taken advantage of the multiple genetic-editing tools available in the *Drosophila* model to assess the mechanisms of innate immune acquisition (Prakash and Khan, 2022). Most studies have used Toll- and Imd-impaired mutants to validate the role of these key pathways in the defense against subsequent infections (Apidianakis et al., 2005; Pham et al., 2007; Aymeric et al., 2010; Christofi and Apidianakis, 2013; Wen et al., 2019; Cabrera et al., 2022). Other studies have used fly lines unable to mount a proper cellular immune response (Christofi and Apidianakis, 2013; Chakrabarti and Visweswariah, 2020), as well as ROS-deficient individuals (Chakrabarti and Visweswariah, 2020). In addition, this model also offers the possibility to study wild populations to assess whether host environment accounted for the difference in resistance patterns observed after infection with the same pathogen (Corby-Harris and Promislow, 2008).

#### Systemic induction of innate immune memory in *Drosophila melanogaster*

Few studies have been performed in *D. melanogaster* addressing this topic (Table 1). Pham et al. (2007) found that the first exposure to a non-lethal dose of *Streptococcus pneumoniae* or to the heat-killed bacteria conferred protection against a second exposure to lethal doses of the same pathogen. This response was found to last the rest of the fly’s life, to be specific and to be mediated by the Toll pathway together with phagocytes, but not the Imd pathway or AMPs (Pham et al., 2007). They also showed that a wide range of heat-killed bacteria, including *S. typhimurium*, *Listeria monocytogenes*, and *Mycobacterium marinum*, which are known to be potent immune activators, did not confer protection to subsequent infections neither with the same pathogen nor with *S. pneumoniae* (Pham et al., 2007). Similar studies were performed with *P. aeruginosa*. Priming flies with the avirulent *P. aeruginosa* strain, CF5, revealed that both Toll and Imd pathways and phagocytosis were necessary for the induction of protection against subsequent infections with the more virulent strain PA14 and that heat-killed CF5 did confer protection although this was shorter (Apidianakis et al., 2005; Christofi and Apidianakis, 2013).

**TABLE 1:** Studies assessing innate immune memory in *Drosophila melanogaster*.

References	Priming species	Challenge species	Time in between	Cell wall component (priming/2nd challenge)	Measures	Evidence of protection	Main results
**Systemic priming**							
Apidianakis et al., 2005	*P. aeruginosa* CF5 (avirulent)	*P. aeruginosa* PA14	3, 6, 12, 24 h	DAP/DAP	Survival and gene expression	Yes	- Best protection 6 h after priming and lasts for >24 h. - Toll and Imd pathways act synergistically to trigger protection.
	Avirulent *E. coli*	*P. aeruginosa* PA14	6 h	DAP/DAP	Survival	Yes	
	Avirulent *S. aureus*	*P. aeruginosa* PA14	6 h	LYS/DAP	Survival	No	
	Avirulent *Cryptococcus neoformans*	*P. aeruginosa* PA14	6 h	β-glucan/DAP	Survival	No	
Pham et al., 2007	*S. pneumoniae* (low dose)	*S. pneumoniae*	1–14 days	LYS/LYS	Survival	Yes	- Priming with *S. pneumoniae* favors survival reducing bacterial load. Protection is long lasting and species-specific. - Phagocytes specifically drives protection to subsequent infection. - Toll pathway is required for the primed response but not Imd.
	hk *S. pneumoniae*	*S. pneumoniae*	1–14 days	LYS/LYS	Survival and CFUs	Yes	
	hk *S. typhimurium*	*S. typhimurium*	7 days	DAP/DAP	Survival	No	
	hk *L. monocytogenes*	*L. monocytogenes*	7 days	LYS/LYS	Survival	No	
	hk *M. marinum*	*M. marinum*	7 days	Mycolic acid	Survival	No	
	*Beauveria bassiana* (low dose)	*B. bassiana*	7 days	β -glucan/β -glucan	Survival	Yes	
	*S. pneumoniae* (low dose)	All the pathogens	7 days	LYS/DAP and LYS	Survival	No	
	Mixture (hk *E. coli, M. luteus, B. bassiana*)	*S. pneumoniae*	7 days	DAP and LYS and β -glucan/LYS	Survival	No	
	Mixture + hk *S. pneumoniae*	*S. pneumoniae*	7 days	DAP and LYS/LYS	Survival	Yes	
Aymeric et al., 2010	Avirulent *E. coli*	*Xenorhabdus nematophila* or *P. luminescens*	1 day	DAP/DAP	Survival and gene expression	Yes	- Increased survival of primed flies 1 day before challenge, but not when coinfected. - Protection mediated by the Imd-related AMPs in the haemolymph previously to challenge
	*P. luminescens* phoP mutant	*P. luminescens*	1 day	DAP/DAP	Survival and gene expression	Yes	
**Systemic priming**							
Christofi and Apidianakis, 2013	*P. aeruginosa* CF5 (avirulent)	*P. aeruginosa* PA14	11 days	DAP/DAP	Survival	Yes	- Short-lasting protective effect - Relish and to a less extend Dif are important for protection. - Immune priming 2 days pre-infection do not depend on phagocytosis. - Immune priming 5 days pre-infection depends heavily on phagocytosis.
	hk *P. aeruginosa* CF5 (avirulent)	*P. aeruginosa* PA14	2, 5 or 7 days	DAP/DAP	Survival	Yes	
Longdon et al., 2013	*Drosophila* C Virus (DCV) (low dose)	DCV (lethal dose)	3 days	–	Survival and viral load	No	- Priming had no effect on flies’ survival. - Mortality correlated with viral presence in the fly.
Kutzer et al., 2019	hk *L. lactis*	*L. lactis*	7 days	LYS/LYS	Survival, CFUs and gene expression	No	- *P. entomophila* priming increased bacterial load at 1 day post-challenge and survival at 28 days post-challenge although non-significantly. - Generally, authors found no effect of the priming on survival, resistance to infection or fecundity - In surviving flies, *L. lactis* becomes a persistent infection but *P. entomophila* is cleared
	hk *P. entomophila*	*P. entomophila*	7 days	DAP/DAP	Survival, CFUs and gene expression	No	
Acuña Hidalgo and Armitage, 2022	hk or CH2O-treated *L. lactis*	*L. lactis*	7 days	LYS/LYS	Survival and CFUs	No	- Priming did not confer any significant advantages to infected flies. - Bacterial load 1 day post-challenge showed bimodal distribution, while 7 days post-challenge was unimodally distributed.
	hk or CH2O-treated *P. burhodogranariea*	*P. burhodogranariea*	7 days	DAP/DAP	Survival and CFUs	No	
Cabrera et al., 2022	*E. faecalis* (low dose)	*E. faecalis*	7 days	LYS7LYS	Survival, CFUs and gene expression	Yes	- Priming with a low dose of *E. faecalis* favors survival at least 7 days later although this increase in survival is not linked with a clearance of the bacteria. - Phagocytosis is needed to mount a primed response to subsequent infection. - Both Imd and Toll pathway are dispensable for the primed response.
**Oral priming**							
Mondotte et al., 2018	DCV (larval stage)	DCV (adult flies)	7–8 days (Transstadial assay)	–	Survival and viral load	Yes	- Priming larvae with DCV showed increased tolerance when challenged in adulthood. - Transstadial immune priming is RNAi-dependent, virus and sequence-specific.
	DCV (larval stage)	Cricket Paralysis Virus (CrPV) and Flock House Virus (FHV) (adult flies)	7–8 days (Transstadial assay)	–	Survival and viral load	Yes	
Patrnogic et al., 2018	Mixture (*E. coli* and *M. luteus*)	*P. luminescens* or *Photorhabdus asymbiotica*	1 day	DAP and LYS/DAP	Survival and gene expression	No	- Imd pathway was upregulated after challenge in flies previously primed with both mixtures.
	Mixture (hk *E. coli* and hk *M. luteus*)	*P. luminescens* or *P. asymbiotica*	1 day	DAP and LYS/DAP	Survival and gene expression	No	- Toll pathway was upregulated after challenge in flies previously primed with live mixture, but not the hk. - Oral priming did not favor survival after challenge.
Wen et al., 2019	hk *E. coli*	*E. faecalis* or *P. aeruginosa*	1 day	DAP/LYS and DAP	Survival and gene expression	Yes	- Gram-positive/Gram-negative-specific protection.
	hk *S. aureus*	*E. faecalis* or *P. aeruginosa*	1 day	LYS/LYS and DAP	Survival and gene expression	Yes	- Priming with hk *S. aureus* showed sexual dimorphism in protection against infection. - Toll and Imd pathways act synergistically to trigger protection.
**Transgenerational priming**							
Bozler et al., 2020	*L. heterotoma* parasitoid wasps (parental flies)	*L. heterotoma* or *L. victoriae* parasitoid wasps (offspring)	Transgenerational assay	–	Survival, blood cells counts and gene expression	Yes	- Longer survival in the offspring of primed parental flies is characterized by enhanced cellular response mainly mediated by a rapid lamellocytes production. - The cellular phenotype in the offspring corresponds with a downregulation of PGRP-LB gene in maternal flies. - There were educed infection rates in the offspring of exposed flies.
Leitão et al. (2020)	*Leptopilina boulardi* parasitoid wasps (parental flies)	*L. boulardi* parasitoid wasps (offspring)	Transgenerational assay	–	Estimation of encapsulation ratio and gene expression	Yes	- The parasitism increased resistance due to a cellular immune system activation which leads to encapsulation over generations.
Mondotte et al., 2020	SINV	SINV	Transgenerational assay	–	Survival, luciferase activity and viral load	Yes	- *D. melanogaster* transmits antiviral immunological memory to their progeny. -Virus and sequence-specific protection, but RNAi independent.
**Others**							
Schwenke and Lazzaro, 2017	Methoprene (synthetic JH analog) (oral)	Hk *Providencia rettgeri* (oral)	–	-/DAP	Survival, CFUs and gene expression	No	- Priming suppressed the induction of AMPs. - JH and Sex Peptide (SP) favor reproduction over protection, suppressing resistance to infection.
Chakrabarti and Visweswariah, 2020	Wound	*E. faecalis* (systemic)	2, 5, or 7 days	-/LYS	Survival and ROS production	Yes	- ROS production in hemocytes is key to activate JAK/STAT and Toll which in turn conferred protection against subsequent infections. - Wound confers short-lasting protection against subsequent infections.
Jacqueline et al., 2020	*P. carotovorum* (oral and cutaneous)	Cancer induction	–	DAP/-	Tumor size and gene expression	Yes	- Infection could have a protective role through the production of Diptericin and Drosomycin that increase tumor cell death in flies primed with *P. carotovorum*, but no with *B. bassiana*.
	*B. bassiana* (oral and cutaneous)	Cancer induction	–	β-glucan/-	Tumor size and gene expression	No	- Toll and Imd pathways act synergistically to trigger protection.
Madhwal et al. (2020)	Wasp-odor food (WOF)	*L. boulard*i parasitoid wasp	–	–	Cellular immune response	Yes	- Those flies pre-conditioned with WOF before infection showed primed their immune response when challenged by elevating their systemic GABA levels which, in turn, promote the pre-differentiation of lamellocytes.
Segrist et al., 2021	Cyclic dinucleotide (CDN) (oral)	DCV or SINV (oral)	0 days	–	Survival and gene expression	Yes	- Oral, but not systemic ingestion of CDNs protect flies against systemic virus infection and induce gene expression in the gut of antibiotic-treated flies. - Protection is dSTING- and dTBK1- dependent. - Toll and Imd pathways act synergistically to trigger protection.

HK, heat-killed, CFUs, colony forming units. CH2O-treated (treated with formaldehyde).

Some authors have evaluated the protection conferred by exposure of *D. melanogaster* to an avirulent *Escherichia coli* against subsequent infection in a short period of time; interestingly, protection on both occasions is driven by the presence of AMPs from the Imd signaling pathway in fly hemolymph prior to challenge (Apidianakis et al., 2005; Aymeric et al., 2010). More recent studies have gone deeper into the mechanisms of these innate immune adaptations. Chakrabarti and Visweswariah defined the ROS production and accumulation in hemocytes after an injury as the key regulators for the induction of the Toll pathway, which in turn confers protection to subsequent infections with *Enterococcus faecalis* (Chakrabarti and Visweswariah, 2020). Nevertheless, this protection conceived by the wound lasted a maximum of 5 days. On the other hand, when *Drosophila* was primed with a low dose of *E. faecalis*, the protection against the second infection with the same pathogen was observed to las for at least 7 days (Cabrera et al., 2022). More insights on the innate immune memory acquisition were described by Tassetto et al. (2017) when *D. melanogaster* was systemically infected with Sindbis virus (SINV). They found that the hemocytes acquired immunological memory in the form of stable virus-derived complementary DNA and that they were able to systemically disseminate it through RNAi-containing exosomes.

Lack of protection after priming has also been documented. For example, systemic injection of killed *Lactococcus lactis*, *Pseudomonas entomophila*, or *Providencia burhodogranariea* did not confer the expected protection against subsequent homologous infections (Kutzer et al., 2019; Acuña Hidalgo and Armitage, 2022); just as systemic infections with a low dose of *Drosophila* C Virus did not protect against subsequent challenge with a lethal one (Longdon et al., 2013). Considering all of the above, protection in *D. melanogaster* is achieved differently depending on the priming agent. This protection seems to be correlated with the immunity pathways activated by this priming species that allows protection, specific or not, against the challenge (Pham et al., 2007; Aymeric et al., 2010; Christofi and Apidianakis, 2013).

#### Oral induction of innate immune memory in *Drosophila melanogaster*

*Drosophila melanogaster* and mammalian intestines are similar both in structure and function, but *D. melanogaster* has a more simple microbiota composed of only 2–30 bacterial species (Wong et al., 2011). However, as seen in mammals, the microbiota can also influence the immune response and shape intestinal function and development in insects (Ryu et al., 2008; Liu et al., 2017; Capo et al., 2019).

Previous research done in which non-pathogenic or heat-killed bacteria were administrated by food to different insects showed, in certain cases, an increased level of AMPs or increased survival. For example, pre-exposure with non-pathogenic *E. coli* can protect larvae of tobacco hornworm *Manduca sexta* from a *Photorhabdus luminescens* infection (Eleftherianos et al., 2006). Moreover, feeding the larvae of cabbage looper *Trichoplusia ni* with non-pathogenic *E. coli* and *Micrococcus luteus* has shown to increase the antibacterial activity (Freitak et al., 2007). These studies serve as a background to establish that microorganisms ingested with the diet, even if non-pathogenic, can trigger an immune response that could reduce the risk of severe disease from other infections.

In *D. melanogaster* oral induction of immune memory has also been assessed (Table 1). Contrary to what happens systematically, a transstadial assay in which larvae were exposed to DCV showed an increase in the tolerance to infections with the same virus in adulthood. This protection was shown to be species-specific as well as RNAi pathway dependent (Mondotte et al., 2018). Specificity of protection was also tested by Wen et al. (2019) by oral priming of *D. melanogaster* with heat-killed bacteria. This study evidenced an heterogeneous protection gram-positive/gram-negative-specific (Wen et al., 2019). In addition, this study also found differences among the levels of AMPs expression between males and females. Females showed higher levels of AMPs after short-term oral priming with killed bacteria, which translated to higher protection against subsequent infection. On the other hand, further research has proven that a previous encounter with non-pathogenic bacteria on the diet failed to protect fruit flies from an infection with entomopathogenic bacteria (Patrnogic et al., 2018). To delve into the mechanisms of fly immunity, priming of flies through diet has also been carried out by administering either suppressors or inducers of the immune response, Juvenile Hormone (JH) or cyclic dinucleotide (CDN), respectively (Schwenke and Lazzaro, 2017; Segrist et al., 2021). Results of these studies showed the impact of the hormonal pathway in *D. melanogaster* in the regulation of the immune response (Schwenke and Lazzaro, 2017), as well as the possibility of bacterial-derived CDNs to induce immunity in microbiota-deficient flies (Segrist et al., 2021).

As another memory mechanism, Madhwal et al. (2020) showed that the systemic elevation of GABA levels neuronally upon olfactory stimulation of *D. melanogaster* (*via* oral), specifically promoted pre-differentiation of lamellocytes and thus, a more efficient cellular response when challenged with *Leptopilina boulardi* parasitoid wasp. Moreover, the protection of *D. melanogaster* by oral priming has not only been proven against subsequent infections. Jacqueline et al. (2020) showed that previous exposure to *Pectobacterium carotovorum* protected flies with a cancer-inducing genotype; results evidenced the importance of both Diptericin and Drosomycin in tumor cell death, and thus, in tumor regression. All these findings suggest that protection provided by an oral exposure to a particular microorganism or antigenic agent is possible in *D. melanogaster*. However, further studies on the mechanisms of immunity are needed in search of more consistent evidence of immunological memory.

#### Transgenerational induction of innate immune memory

It has also been described in insects that innate immune memory can pass down from primed parent individuals to the offspring (Dhinaut et al., 2018; Vilcinskas, 2021). This kind of memory is currently named as Transgenerational Immune Priming (TgIP) and has been studied in a wide range of insects, although only a few used the *Drosophila* model. However, the exact mechanism is not yet fully understood, as well as how many generations it lasts (Sheehan et al., 2020). One of the possible mechanisms is the transgenerational transfer of microbial elicitors, in which bacteria or bacterial fragments ingested by female individuals are translocated to their eggs following the same route as bacterial symbionts, thus providing the offspring with the capacity to mount a more specific innate immune response against these bacteria (Herren et al., 2013; Freitak et al., 2014; Knorr et al., 2015). However, some studies also provide evidences of paternal TgIP in insects which are thought to be induced by epigenetic modifications transferred to the germ cells and transmitted to the offspring (Soubry et al., 2014; Zenk et al., 2017).

Using the *D. melanogaster* model, Bozler et al. (2020) showed that increased maternal Peptidoglycan Recognition Protein LB (PGRP-LB) expression levels after exposure to parasitic wasp correlated with a more successful immune response to the parasite in the offspring mainly mediated by a rapid cellular activation. Same implication of the cellular immune response in TgIP was observed in flies primed with another parasitic wasp species (Leitão et al., 2020). Mondotte et al. (2020) also described an antiviral transgenerational immune priming in both *D. melanogaster* and the mosquito *Aedes aegypti* after parental priming with different single stranded RNA viruses with specificity in the protection of the progeny for several generations. On the contrary, it has also been seen how the offspring inherited indirect costs associated with the immune response to the infection of the progenitors, having shorter lifespans (Linder and Promislow, 2009).

#### Measuring immune memory readouts: A *Drosophila melanogaster* approach

Once flies are primed, measurement of tolerance and resistance to the subsequent infection is needed to assess the acquisition of innate immune memory. In this regard, different theoretical models have been used along time (Råberg et al., 2008; Lefèvre et al., 2011; Gupta and Vale, 2017). The application of these models is not absolute and aspects such as the experimental model used, its lifespan, or the possibility of carrying out a certain test along the course of infection, will determine which model is the most appropriate at each moment. In the literature, resistance is defined as the inverse of the pathogen concentration (number of parasites per host or per unit tissue); when all the other variables are equal, a lower Y-intercept means the host is more resistant. Tolerance, on the contrary, is usually defined as the slope of a linear regression model when plotting host fitness against infection intensity in 2- dimensional health-by-microbe space; the flatter the slope, the higher the tolerance (Råberg et al., 2008). If the slope varies among groups, such that the fitness of some hosts declines faster with increasing inoculation doses, this means there is variation in tolerance among hosts types (Råberg et al., 2008; Ayres and Schneider, 2012).

In ecology and evolutionary biology, this description of how specific individuals respond to different environmental conditions is known as the “reaction norm” and considers the different pathogen burdens that can be applied to infections (Råberg et al., 2007). Depending on the animal model, host fitness can be measured differently. In mammals, the most often measured effects of infection on host health are anemia and weight loss and they are plotted against the peak pathogen density (Råberg et al., 2008). In insects, counting the Bacillary Load Upon Death (BLUD, in case of bacterial infections) is interesting as dead caused by disease is correlated with the ability of the pathogen to proliferate and the ability of the host to react against that infection. Therefore, as explained, the maximal bacterial load that flies can cope with before death represents a measure of host disease tolerance (Duneau D. et al., 2017). Moreover, the Set Point Bacterial Load (SPBL), meaning measuring the colony forming units (CFUs) at different time points, is also a useful observation to dissect the mechanism taking place throughout the course of infection. Studies done with *D. melanogaster* aim to measure bacillary load against inoculation dose as a measure of resistance (Corby-Harris et al., 2007; Kutzer et al., 2019; Wen et al., 2019), and both host’s health or fecundity as the host-fitness parameter against inoculation dose to measure tolerance (Kutzer et al., 2019).

When using this linear model, four different patterns could be observed. A low survival with low pathogen load could explain the metabolic costs associated with mounting an immune response. When a high mortality is associated with a high bacterial load, it might be due to the high costs of fighting a virulent microorganism. High survival rates associated with low pathogen loads suggest that a robust immune response is present, and the host is resistant to the infection. In contrast, a high survival with high bacterial loads suggests a tolerant phenotype in which the host can survive longer since it does not need to use sources to clear the infection (Corby-Harris et al., 2007; Kutzer and Armitage, 2016; Duneau D. et al., 2017; Box 2). In contrast to the linear models, Gupta and Vale proposed a 4-parameter non-linear model in *D. melanogaster* to estimate disease tolerance with more detail (Gupta and Vale, 2017). While linear approaches bring light to the rate at which hosts lose health, non-linear approaches are useful to clarify the dose that causes this lost in health or the severity of the infection (Gupta and Vale, 2017).

**Box 2. Patterns associated with an infection according to linear models.**

**Table:** 

Metabolic cost of immunity	Low survival and low pathogen load.
Insufficient immune response against a virulent microorganism	Low survival and high pathogen load.
Resistant phenotype	High survival and low pathogen load.
Tolerant phenotype	High survival and high pathogen load.

## Discussion

Over the years, scientists have used the *D. melanogaster* model to study innate immunity. However, the mechanisms behind the induction of innate immune memory in this host model are still not fully elucidated. Previous research has shown that a former encounter of *D. melanogaster* with heat-killed, not pathogenic bacteria or low dose of pathogens could confer protection from future infections with pathogenic microorganisms (Pham et al., 2007; Cooper and Eleftherianos, 2017; Patrnogic et al., 2018).

Interestingly, this mechanism cannot be generalized, so certain parameters have to be standardized first to have a better comparison of the results. Susceptibility to infection in flies can vary according to age, sex, genetics, feeding and physiological and environmental factors, such as temperature, resources, humidity, time of the day or light; or even according to the presence of other infections or single-nucleotide polymorphisms (SNPs) that could confer greater vulnerability to certain pathogens (Merkling and Van Rij, 2015; Duneau D. F. et al., 2017; Kutzer et al., 2019; Troha and Buchon, 2019). However, the advantage of this model is that experiments can be repeated easily and in an affordable manner. Such a quantity of factors regulating fly immune response explains why not every challenge with a non-lethal dose of a pathogen induces protection from further encounters, and why the duration of this protection is variable (Hillyer, 2016; Hajishengallis et al., 2019), although it was suggested that it might pass to the progeny (Mondotte et al., 2020). The differences on the kinetics of the different immune pathways that are activated upon infection, as well as the sexual dimorphism that exists when mounting an immune response, also influence the acquisition of innate immune memory. In addition, a different pattern of activation is observed between sexes (Duneau D. F. et al., 2017; Belmonte et al., 2020), which translate with different protection levels against a second exposure to the pathogen (Wen et al., 2019).

As tolerance and resistance confer protection to a host at different levels when infected, several models to measure their acquisition have been described (Råberg et al., 2008; Lefèvre et al., 2011; Gupta and Vale, 2017). Nevertheless, the choice of the model will depend such as the nature of the host or the answers we want to obtain. Recent research on this topic pointed out that tolerance and resistance mechanisms can have a differential importance throughout the course of the infection. For example, during the early phases of *Listeria monocytogenes* infection in mice, resistance mechanisms were predominant, whereas tolerance was more important during the last stages. This also points out the fact that both mechanisms are interconnected (Kutzer et al., 2019). Examples of immune priming that could protect insects from future infections were seen when the larvae of wood tiger moth *Parasemia plantaginis* received a diet containing a low dose of *Serratia marcescens* or non-pathogenic *E. coli*. Larvae primed with *S. marcescens* were later protected from an otherwise lethal injection of the same pathogen. This, however, was not the case of larvae primed with *E. coli*. This study suggests that pathogen recognition at the midgut level can be important for conferring systemic immunity (Mikonranta et al., 2014). Moreover, other examples include the oral administration of peptidoglycans from the cell wall of *P. aeruginosa*, or heat-killed *P. aeruginosa* conferred protection against a following infection with *P. aeruginosa* in silkworms. However, silkworms receiving heat-killed *S. aureus* did not show an increased survival after infection with *P. aeruginosa* (Miyashita et al., 2015). This review underlines the lack of standardized method for assessing the type of protection provided by priming the host with different stimuli, as each author focused on different parameters, such as survival, pathogen load or gene expression. However, the assessment of all three parameters would be of interest in order to define the mechanism behind innate immune memory.

Immune priming in *D. melanogaster* has been studied on several occasions. And, although the results verify the great variability that the fly presents in terms of protection, they allow us to obtain an increasingly precise overall view of the mechanisms behind trained immunity. Most of the studies conclude that a certain specificity between the priming pathogen, the innate immune response triggered by the host and the protection that this provides against subsequent infections. This specificity has been observed at species level (Pham et al., 2007) and sequence-specific in viruses (Mondotte et al., 2020). Fewer studies have also shown less specific protection e.g., between Gram-negative/Gram-positive species (Wen et al., 2019). Nevertheless, in those studies evidencing protection against subsequent infections is not easy to discriminate if this protection is due to innate immune memory acquisition or due to the fact that the immunity is still stimulated in the flies at the time of the second challenge (Chambers et al., 2019). Further studies which test maximum times in between priming and challenge, as well as the maximum duration of protection would help to elucidate this. In addition, it would be interesting to perform more priming tests in this animal model to further elucidate the specificity of protection and the ability to transmit this protection to offspring. In addition, oral priming prior to the second challenge has been poorly studied in this experimental model. A more exhaustive study of the subject would be interesting in order to screen protective components against infection with the possibility of further translation to other animal models. The use of *Drosophila* to assess oral priming might be very useful considering the possibility to study local adaptation of wild-caught strains, which are in constant contact with different pathogen-rich environments (Corby-Harris and Promislow, 2008).

Bearing all of the above in mind, it is still a challenge to decipher innate immune memory mechanisms induced after infection with a pathogen and further research is needed. As selecting the ideal model is crucial for future developments, non-mammalian models, especially *D. melanogaster*, are a good alternative to deep into its study.

## Author contributions

All authors listed have made a substantial, direct, and intellectual contribution to the work, and approved it for publication.
